# *Lactobacillus*-derived extracellular vesicles enhance host immune responses against vancomycin-resistant enterococci

**DOI:** 10.1186/s12866-017-0977-7

**Published:** 2017-03-14

**Authors:** Ming Li, Kiho Lee, Min Hsu, Gerard Nau, Eleftherios Mylonakis, Bharat Ramratnam

**Affiliations:** 1 Division of Infectious Diseases, The Warren Alpert Medical School of Brown University, Providence, RI 02903 USA; 2 COBRE Center for Cancer Research, Rhode Island Hospital, Warren Alpert Medical School of Brown University, Providence, RI 02903 USA; 3 Clinical Research Center of Lifespan, Providence, RI 02903 USA

**Keywords:** *Lactobacillus*, Extracellular vesicles, LDEVs, Vancomycin-resistant enterococci, VRE, *CTSB*, *REG3G*

## Abstract

**Background:**

Probiotic bacteria are known to modulate host immune responses against various pathogens. Recently, extracellular vesicles (EVs) have emerged as potentially important mediators of host-pathogen interactions. In this study, we explored the role of *L. plantarum* derived EVs in modulating host responses to vancomycin-resistant *Enterococcus faecium* (VRE) using both *Caenorhabditis elegans* and human cells.

**Results:**

Our previous work has shown that probiotic conditioning *C. elegans* with *L. acidophilus* NCFM prolongs the survival of nematodes exposed to VRE. Similarly, *L. plantarum* WCFS1 derived extracellular vesicles (LDEVs) also significantly protected the worms against VRE infection. To dissect the molecular mechanisms of this EV-induced protection, we found that treatment of *C. elegans* with LDEVs significantly increased the transcription of host defense genes, *cpr-*1 and *clec-60*. Both *cpr-*1 and *clec-60* have been previously reported to have protective roles against bacterial infections*.* Incubating human colon-derived Caco-2 cells with fluorescent dye-labeled LDEVs confirmed that LDEVs could be transported into the mammalian cells. Furthermore, LDEV uptake was associated with significant upregulation of *CTSB*, a human homologous gene of *cpr-1,* and *REG3G*, a human gene that has similar functions to *clec-60*.

**Conclusions:**

We have found that EVs produced from *L. plantarum* WCFS1 up-regulate the expression of host defense genes and provide protective effects on hosts. Using probiotic-derived EVs instead of probiotic bacteria themselves, this study provides a new direction to treat antimicrobial resistant pathogens, such as VRE.

**Electronic supplementary material:**

The online version of this article (doi:10.1186/s12866-017-0977-7) contains supplementary material, which is available to authorized users.

## Background

*Lactobacillus* is a genus of Gram-positive facultative anaerobic bacteria [1]. Considered as non-pathogenic and generally regarded as safe, lactobacilli have been widely used for fermentation and food production for centuries [2, 3]. The beneficial or probiotic effects of lactobacilli are under intense investigation with both laboratory and clinical studies [4–8], suggesting that administration of lactobacilli inhibit cytokine-induced apoptosis [9] and decreases the pathogenicity of various pathogens, such as *E. coli* [10] and VRE [11]. However, the molecular mechanisms by which lactobacilli impact VRE are incompletely understood.

Lactobacilli may exert immunomodulatory effects using multiple mechanisms including binding directly to C-type lectin receptors (CLRs) or Toll-like receptors (TLRs), on the host cell surface [12, 13]. For example, administration of *L. casei* CRL 431 increased the expression of TLR2, TLR4, and TLR9 and improved the production and secretion of TNFα, IFNγ, and IL-10 in mice [12]. Alternatively, lactobacilli may produce antimicrobial substances to inhibit the growth of various pathogens. For example, a bacteriocin produced by lactobacilli formed pores in the membranes of pathogens and thus caused leaking of target cells [14, 15]. More recently, studies have revealed that extracellular vesicles (EVs) and associated proteins from lactobacilli can also modulate the activity of immune cells and affect host innate and adaptive immune responses [16–18]. For example, EVs from lactobacilli were found to enhance cellular TLR2/1 and TLR4 responses while suppressing TLR2/6 signaling [17].

Extracellular vesicles (EVs) are nanometer-scale membrane-contained vesicles released in an evolutionally conserved manner by a wide range of cells [19, 20]. By facilitating the transfer of proteins, nucleic acids, and other molecules between cells [21, 22], EVs are associated with molecular transport, mediation of stress response and biofilm formation thus influencing their hosts [23, 24]. This EV-mediated interaction is likely prevalent in the gut as a major method of communication between bacteria and hosts, since a layer of mucin prevents direct physical contact between bacteria and host tissues [25]. Another unique feature associated with EVs is their potential to mediate therapeutic molecule delivery without inducing adverse immune reactions [26].

In this study, we selected *L. plantarum* WCFS1, a leading probiotic strain found in the gastrointestinal tract, due to its potency to inducing immunomodulatory effects [27]*.* We found that *L. plantarum* WCFS1 produces EVs that are 30–200 nm in diameter. Proteomic analysis revealed that *L. plantarum* derived EV (LDEV) cargo was enriched with membrane-associated proteins. Using the experimental nematode *C. elegans*, LDEV treatment prolonged the survival rates of *C. elegans* under *E. faecium* (VRE) challenge*.* To investigate the underlying mechanisms, we found that the host defense genes, *cpr-1* and *clec-60*, were significantly upregulated. LDEV treatment of human colonic cells lines also led to similar upregulation of *CTSB* (Cathepsin B) and *REG3G* (Regenerating islet-derived protein 3-gamma).

## Results

### *L. plantarum* produces EVs

**Fig. 1:**
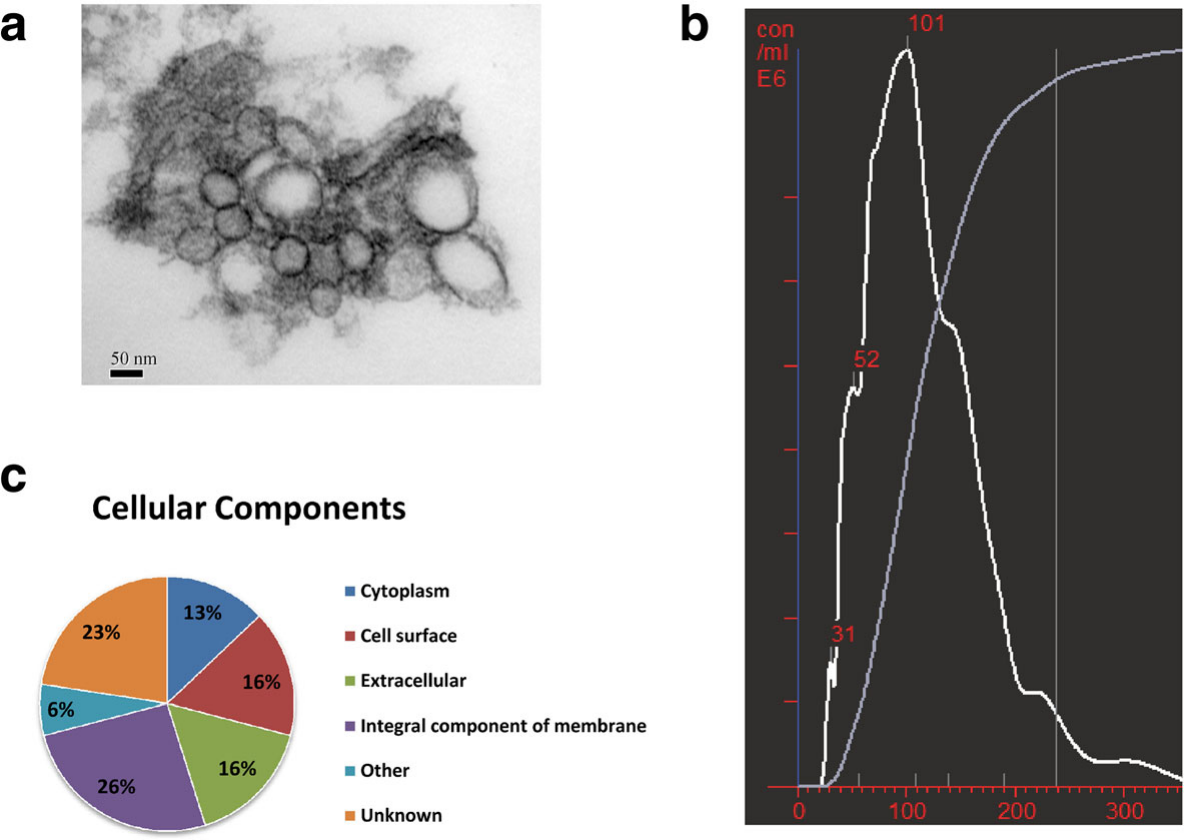
**a** Electron microscopy of *L. plantarum* WCFS1 EVs. Representative transmission electron micrograph shows EVs isolated from *L. plantarum* growth medium, magnification 92,000. EVs measure between 30 and 150 nm in diameter and have the morphologic appearance consistent with EVs. **b** NanoSight size analysis of *L. plantarum* WCFS1 EVs. The graph represents the size (X-axis) versus concentration (Y-axis) where the white line represents EV size distribution, and the gray line is the accumulated percent of EVs assayed. Over 80% of EVs are sized between 31 nm and 200 nm, while the highest enriched EVs are around 101 nm. **c** Gene ontology analysis of *L. plantarum* WCFS1 EV proteome. Eighteen out of thirty-one proteins were found to be part of membrane or associated with membrane, where typical bacterial EVs either get produced or exported

We isolated EVs from the supernatant of *L. plantarum* WCFS1 using ExoQuick-TC kit (System Biosciences) [28]. The isolated particles were characterized by electron microscopy, nanoparticle tracking analysis (NTA, NanoSight) and proteomic identification. Electron microscopy showed typical EV-like size (30–200 nm) and morphologic appearance (enclosed by membranes) (Fig. 1a). NTA analysis, a measure of particle size, revealed that over 80% of isolated EVs ranged from 31 nm to 200 nm (Fig. 1b), within the range of previously described EV sizes between 30 and 1000 nm [23]. We also used liquid chromatography–mass spectrometry to profile EV protein content. 31 proteins were identified in the EV fraction (Additional file 1: Table S1). Notably, according to gene ontology analysis, over half of the proteins were found to be associated with membrane, where typical bacterial EVs originate (Fig. 1c) [29, 30]. In all, these results confirmed that *L. plantarum* WCFS1 produces and release EVs.

### *L. plantarum* EVs are biofunctional and increase the survival of *C. elegans*

**Fig. 2:**
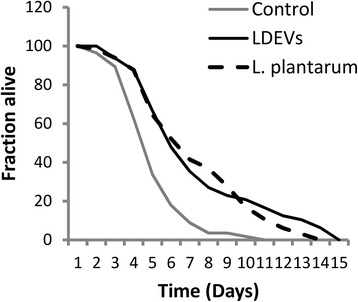
Conditioning with LDEVs prolonged the survival of *C. elegans* nematodes infected with VRE. Compared to the worms conditioned with mock EVs, significantly longer survival was found in the LDEV conditioned worms (~4 days, *p* < 0.01). *L. plantarum* WCFS1 conditioned worms also had significantly (~3 days, *p* < 0.01) longer survival than those conditioned with mock EVs. There was no statistical difference in survival between worms conditioned with LDEVs and with *L. plantarum* WCFS1 bacteria

Our previous study showed that preconditioning *C. elegans* with *L. acidophilus* NCFM prolongs the survival of the nematode after infection with *Enterococcus faecalis* [31]. We asked if EV fractions of lactobacilli can also provide similar protective effects. Using an agar-based solid killing assay [32], *C. elegans* were pre-treated with *L. plantarum* bacteria, LDEVs or mock EV’s as described in the Methods section. The nematodes were then challenged with a clinically isolated vancomycin-resistant *E. faecium* C68. Compared to the control worms conditioned with mock EVs, *C. elegans* conditioned with *L. plantarum* WCFS1 bacteria survived significantly longer (~3 days) (Fig. 2). This result is similar to that previously obtained using *L. acidophilus* [31]. Notably, worms pre-treated with LDEVs also survived significantly longer (~4 days) than those treated with mock EVs. We did not observe significantly different survival between LDEV treated and *L. plantarum* treated groups (Fig. 2)*.*

### *L. plantarum* EVs up-regulate host defense genes, *clec-60* and *cpr-1* in a *C. elegans* model

**Fig. 3:**
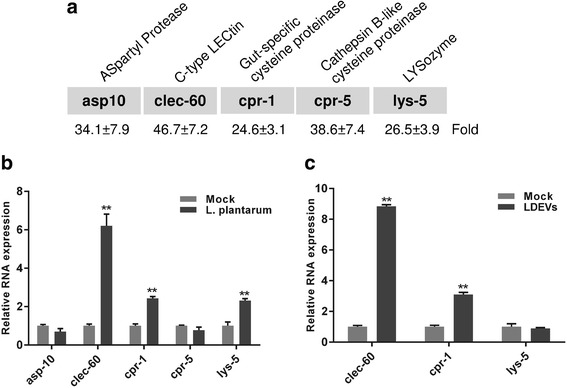
Expression profiles of host defense genes when *C. elegans* were conditioned with lactobacilli and then LDEVs. **a** RNAs of five host defense genes (*asp10, clec-60, cpr-1, cpr-5*, and *lys-5*) were significantly (*p* < 0.01) up-regulated when *C. elegans fer-15;fem-1* were conditioned with *L. acidophilus* NCFM (See reference [31]). Data are derived from qPCR with fold change in gene expression listed below each gene. **b** RNAs of *clec-60*, *cpr-1* and *lys-5* were significantly (**, *p* < 0.01) up-regulated when *C. elegans fer-15;fem-1* were conditioned with *L. plantarum* WCFS1. **c** Significant (**, *p* < 0.01) up-regulation of *clec-60* and *cpr-1* was associated with LDEV conditioned *C. elegans fer-15;fem-1*

The protection induced by LDEVs prompted us to investigate the possible immunomodulatory effects of LDEVs on *C. elegans*. Our previous research had shown that five host defense genes (*asp10, celec-60, cpr-1, cpr-5,* and *lys-5*) were significantly up-regulated when *C. elegans* were conditioned with *L. acidophilus* NCFM (Fig. 3a) [31]. When *L. plantarum* bacteria were applied to *C. elegans*, a similar significant up-regulation of *clec-60* (~6.2 fold), *cpr-1*(~2.4 fold) and *lys-5*(~2.3 fold) was observed (Fig. 3b). When *C. elegans* were treated with *L. plantarum* derived EVs, we observed a significant up-regulation of gene expression for the C-type lectin *clec-60* (~9 fold) and the gut-specific cysteine protease *cpr-1*(~3 fold) (Fig. 3c).

### *L. plantarum* EVs incubation led to LDEV cargo delivery and up-regulation of host defense genes, *CTSB* and *REG3G* in Caco-2 cells

**Fig. 4:**
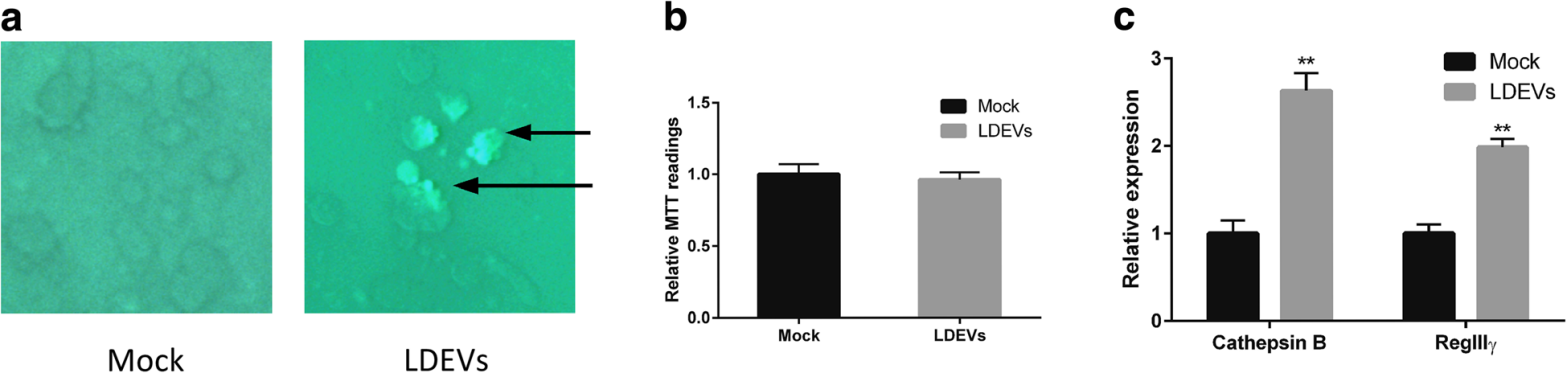
**a** The incubation of LDEVs with Caco-2 cells led to cargo delivery from LDEVs to Caco-2 cells. Compared to mock EVs, only LDEVs treated Caco-2 group showed positive fluorescence. **b** The incubation of LDEVs did not cause any significant toxicity to Caco-2 cells. MTT assay was employed to examine the viability of Caco-2 cells 24 h after they were treated with mock EVs or LDEVs. **c** LDEVs increased gene expression of *CTSB* and *REG3G* in Caco-2 cells. At 24 h post-incubation, the RNA expression of *CTSB* (2.5-fold, *p* < 0.01) and *REG3G* (2-fold, *p* < 0.01) were significantly up-regulated in the LDEV treated group

Having established the immunomodulatory effects of LDEVs in the nematode model, we next investigated the impact of LDEVs on Caco-2 cells as a model of human colonic epithelium [33]. LDEVs were fluorescently labeled with Exo-Green and then incubated with Caco-2 cells for 24 h. We observed ~25% of Caco-2 cells retained the fluorescent label after washing. No detectable fluorescence was observed from the mock EV control, which went through the same EV isolation and labeling procedures (Fig. 4a). Incubating LDEVs with Caco-2 cells did not impact the viability of the mammalian cells (Fig. 4b). Next, we tested if the two genes that showed significantly increased expression in *C. elegans*, *clec-60,* and *cpr-1*, translated to the mammalian model system. CTSB [34], the cysteine proteinase, is the human orthologue of *cpr-1*. There is no direct human orthologue of *clec-60* based on sequence homology. We, therefore, investigated REG3G*,* an intestinally secreted C-type lectin that likely has functional similarity [35, 36]. Both *CTSB* and *REG3G* RNAs were significantly up-regulated in Caco-2 cells after the LDEV treatment (Fig. 4c). This upregulation of *CTSB* and *REG3G* confirmed the results obtained from *C. elegans* model.

## Discussion

The importance of EVs has been increasingly recognized. Virtually all kinds of cell types studied so far secret EVs, and they are also found in various bio-fluids [19, 20]. This phenomenon indicates that EVs are evolutionarily conserved and likely functionally important [21, 22]. Indeed, numerous studies on mammalian cell derived EVs have shown that EVs play important roles in intercellular communication and mediation of immunomodulatory response [22]. However, EV-mediated interactions between host and bacterial pathogens are less explored. Limited studies suggest that pathogenic bacterial strains affect biofilm formation via EV pathways [23, 24]. A recent study on probiotic bacteria has also shown EVs from multiple *Lactobacillus* strains modulate host-microbe responses by regulating the TLR2 activity and phagocytosis [17]. Here, we focused on *L. plantarum*, a gut-associated commensal bacteria often used in probiotic nutritional supplements. We found that *L. plantarum* WCFS1 produces functional EVs that enhance host defense gene expression and directly augments protection against VRE infections. These findings suggest LDEVs, at least partially, mediate the immunomodulatory properties of probiotic lactobacilli.

It is interesting to note that *L. plantarum* derived EVs up-regulate *clec-60* and *cpr-1*, while the *L. plantarum* bacteria promote the expression of both genes and *cpr-5*. The shared upregulation of *clec-60* and *cpr-1* suggest that *L. plantarum* derived EVs retain much of the immunomodulatory effects of *L. plantarum.* This is probably because EVs have similar cargo contents as their parental bacteria [23]*.* The different regulation observed with gene *cpr-5*, however, illustrates that bacterial EVs are not equal to the intact bacteria regarding the spectrum of induced immunomodulatory effects.

Our experiments using human Caco-2 cells confirmed biological activity of the LDEVs. Both *REG3G* [36], which is functionally similar to *clec-6*, and *CTSB* [37] (the human orthologue of *cpr-1*) are upregulated by LDEV treatment. REG3G is an intestinally secreted C-type lectin with potent bactericidal activity against Gram-positive bacteria [35]. It also promotes the spatial segregation of microbiota and host in the intestine [36], thus decreasing the chance of bacterial colonization on the intestinal epithelial surfaces [38, 39]. CTSB, a cysteine proteinase involved in cell death and inflammation [40], is associated with antibacterial activity [41, 42]. Although it may involve autophagy [43], the exact mechanism of CTSB on bacterial pathogens is unclear.

This study provided a mechanistic insight as to how LDEVs enhance host immune response via upregulation of the two host genes, *REG3G* and *CTSB*.

## Conclusions

In summary, our study revealed that EVs produced from *L. plantarum* up-regulate the expression of host defense genes, *clec-60* and *cpr-1,* and provide protection against VRE infection in a *C. elegans* model. LDEV treatment of human colonic cells lines also led to similar upregulation of *CTSB* and *REG3G*. The findings of this study could be harnessed to design a new therapeutic treatment of antimicrobial resistant infections by using EVs derived from probiotic strains rather than the bacteria themselves.

## Methods

### Preparation of probiotic bacteria

Single colony inoculated *L. plantarum* WCFS1 (BAA-793, ATCC) was grown in de Man, Rogosa, and Sharpe (MRS) medium (Difco Laboratories) at 37 °C for 24 h.

### Isolation of extracellular vesicles

Extracellular vesicle fractions were independently enriched from culture supernatants of *L. plantarum* WCFS1 and medium control. Supernatants from overnight cultures were generated by first centrifuging cultures at 1000 g for 10 min. All supernatants were then passed through a 0.22 μm filter to remove large particles and possible contaminants. EVs were isolated using an ExoQuick-TC™ (System Biosciences) kit per the manufacturer’s directions. Briefly, five parts of supernatant were mixed with one mL of ExoQuick-TC solution. The mixtures were incubated overnight at 4 °C and followed by two centrifugations at 1500 × g for 30 min and then 5 min, respectively. The supernatants were discarded, and the resulting pellets were resuspended in PBS to use directly in downstream experiments or placed in a −80 °C freezer for long-term storage. Mock EVs were isolated from sterile, uninoculated *L. plantarum* WCFS1 culture broth using the same EV isolation procedures.

### Electron microscopy

LDEVs were fixed with 3% glutaraldehyde in 0.15 M sodium cacodylate buffer and then post-fixed in 1% osmium tetroxide (Electron Microscopy Sciences). Fixed samples were cut into 1.5 mm cubes and covered with a 3% agar solution. Samples were dehydrated through a graded series of acetone and embedded in Spurr epoxy resin (Ladd Research Industries). Ultra-thin sections were then prepared, retrieved onto 300-mesh thin bar copper grids, and contrasted with uranyl acetate and lead citrate. Sections were examined using a Morgagni 268-transmission electron microscope and images collected with an AMT Advantage 542 CCD camera system.

### Nanoparticle tracking analysis (NTA)

The NTA analysis was carried out using a NanoSight™ NS500 (Malvern) and an automatic syringe pump system. This instrument generates a detailed analysis of the size distribution and concentration of nanoparticles. The analysis was performed on EVs suspended in PBS at 22 °C. Thirty of 30-s videos were recorded for each sample with camera shutter at 33 ms. Videos recorded for each sample were analyzed with NTA software (version 2.3). For this analysis, auto settings were used for blur, minimum track length, and expected particle size; detection threshold was set at 4 Multi.

### Proteomics

Proteomic characterization of LDEVs was performed by liquid chromatography-tandem mass spectrometry (LC-MS/MS, nano-LC from LC Packings/Dionex, and Qstar XL from Applied Biosystems). LDEV samples were suspended in Novex® (Thermo Scientific) reducing sample buffer and heated for 10 min at 70 *°*C. Samples were run on Novex® 4-20% Tris-Glycine gradient gels and stained with SimplyBlue® SafeStain (Life Technologies) for 1 h followed by destaining with water. Gel bands were excised and digested with modified Trypsin (Promega). Tryptic digests were fractionated with a reversed-phase column and the column eluate introduced onto a Qstar XL mass spectrometer via ESI. Protein identifications were performed with ProteinPilot (Applied Biosystems) using the *L. plantarum* WCFS1 reference sequence database from UniProt and NCBI. To increase confidence, a further manual inspection was carried out to select the proteins associated with at least two unique as the potential candidates [44–46].

### Gene ontology (GO) analysis

Protein candidates listed in Additional file 1: Table S1 were searched against UniProt, EBI, and GO databases. Visualization of GO analysis results was carried out in Excel.

### Nematode and pathogenic bacteria

*C. elegans* Bristol N2 *or fer-15;fem-1* was used in this study. *C. elegans* strains were routinely maintained on nematode growth medium (NGM) plates seeded with *E. coli* OP50 or HB101 using standard procedures [47]. Clinically isolated *Enterococcus faecium* (vancomycin-resistant) C68 [48] was grown at 37 °C using brain heart infusion (BHI; Difco) broth.

### *C. elegans* killing assays

Solid killing assays were performed using published methods, with slight modifications [47]. For positive control, 1x10^9^ CFU *L. plantarum* bacteria were spread on an SK plate. LDEVs that were isolated from an equivalent number of *L. plantarum*, 1x10^9^ CFU, were suspended in PBS and spread on an SK plate. For the negative control, the same volume of mock EVs was spread on SK plate. Each plate was dried for 3 h at room temperature before use. Forty to sixty *C. elegans* Bristol N2 were seeded into each plate after pre-incubating with *L. plantarum* WCFS1, LDEVs or controls for 24 h, followed by VRE challenge (a clinically isolated C68 *E. faecium* strain at 1x10^9^ VRE/plate). After worms had been placed on the plates with the VRE, plates were then incubated at 25 °C and examined for viability at 24-h intervals for 15 days using a Nikon SMZ645 dissecting microscope. Worms were counted as alive or dead based on their response or lack of response to gentle touching with a platinum wire. For preventing hatching of examined adult worm, worms were treated with 5-fluorodeoxyuridine (50 μM) from L4 to end of assays.

### Culture and EV treatment of cell lines

Caco-2 (HTB-37, ATCC), a human colon carcinoma cell line, was maintained in Eagle’s Minimum Essential Medium (EMEM) supplemented with 20% fetal bovine serum (FBS) and was used to test LDEV’s effect on mammalian cells. LDEVs were labeled by Exo-Green (System Biosciences) according to manufacturer’s instruction. Briefly, 500 μl of LDEVs suspended in PBS was mixed with 50 μl stain. After 10 min 37 °C incubation and precipitation by ExoQuick-TC, the labeled LDEVs was re-suspended in PBS and added to Caco-2 cells. At 24 h post-incubation, the culture wells were rinsed twice with PBS to remove residual fluorescent dyes. The cells were then examined by fluorescent microscopy (Olympus IX-70). A control experiment using mock EV was also carried out in parallel.

### Caco-2 viability assay

(3-[4,5- dimethylthiazol-2-yl]-2,5-diphenyl tetrazolium bromide) or MTT assay (Sigma) was used to measure Caco-2 cellular proliferation rate after LDEV treatment. All procedures were performed according to the manufacture’s instruction.

### RNA isolation and qPCR

Total RNA from *C. elegans* and Caco-2 cells was extracted using TRIzol® (Thermo Scientific) by following standard protocols. The concentrations of all RNA samples were determined by spectrophotometry. 1 μg of total RNAs was used for reverse transcription and PCR, which was carried out on a Mastercycler® gradient 5331 (Eppendorf, Westbury, NY) by using Maxima® First Strand cDNA Synthesis Kit (Thermo Scientific). Primers were designed by using PrimerQuest online tools available at http://www.idtdna.com/Primerquest/Home/Index. Primer sequences are provided in Additional file 2: Table S2. Real-time PCR was performed on Mastercycler® ep realplex (Eppendorf). All reactions were performed in 96-well plates with the following reagents in a final volume of 20 μl: 1 μl of primers (5 μM each for forward and reverse) and 2X Maxima® SYBR Green qPCR Master Mix from Thermo Scientific. 10 ng of cDNA was added to this mixture. Triplicate reactions of the target and housekeeping genes were performed simultaneously for each cDNA template analyzed. The PCR reaction consisted of an initial enzyme activation step at 95 °C for 10 min, followed by 40 cycles of 95 °C for 15 s and 60 °C for 1 min. A cycle threshold value (Ct) value was obtained for each sample, and triplicate sample values were averaged. The 2^-ΔΔCt^ method was used to calculate relative expression of each target gene. The control genes *snb-1* [49] and *ACTB* [50] were used to normalize the gene expression data from *C. elegans* or Caco-2 cells respectively.

### Statistical analysis

The log-rank test was used to determine the difference in *C. elegans* survival rates. Differences in qPCR results were determined by using the Student’s t-test. A *P* < 0.05 in all experiments was considered statistically significant. Statistical analysis and graphing were performed with Prism version 6.05 (GraphPad).

## Additional files

Additional file 1: Table S1. Proteomics data from L. plantarum derived extracellular vesicles. Protein name, GO term (cellular component), Accession number, Peptide sequence, Validation Score, Xcorr score, Change in mass (ppm), Isolated mass, Peak area, Charge state, and Number of peptides are given. (XLSX 28 kb)
Additional file 2: Table S2. qPCR primers. Their names and sequences are given. (XLSX 8 kb)

## Abbreviations

CTSB: Cathepsin B
EVs: Extracellular vesicles
IFNγ: Interferon gamma
IL-10: Interleukin 10
LC-MS/MS: Liquid chromatography–tandem mass spectrometry
LDEVs: *L. plantarum* derived extracellular vesicles
qPCR: Quantitative polymerase chain reaction
REG3G: Regenerating islet-derived protein 3 gamma
TLR: Toll-like receptor
TNFα: Tumor necrosis factor alpha
VRE: Vancomycin-resistant enterococci
